# Prevalence of scrotum bipartition in sheep in the Paraiba backwoods, Brazil

**DOI:** 10.21451/1984-3143-AR2018-0071

**Published:** 2018-12-05

**Authors:** Ediane Freitas Rocha, Rômulo Freitas Francelino Dias, Nayadjala Távita Alves dos Santos, Lamartine José Brito Medeiros, José Rômulo Soares dos Santos, Severino Silvano dos Santos Higino, Maria Acelina Martins de Carvalho, Otávio Brilhante de Sousa, Sérgio Santos de Azevedo, Danilo José Ayres de Menezes

**Affiliations:** 1 Universidade Federal de Campina Grande, Patos, PB, Brazil.; 2 Universidade Federal do Piauí, Teresina, PI, Brazil.; 3 Universidade Federal do Rio Grande do Norte, Natal, RN, Brazil.

**Keywords:** morphology, animal production, ruminants, semi-arid

## Abstract

Goats and sheep have morphological characteristics for adaptation to desert and semiarid regions. The appearance of scrotum division known as scrotum bipartition has already been reported in goats. This anatomy increases the surface of each testicle exposed to environmental temperature, favoring heat dissipation and improving reproductive efficiency. Considering that there are already studies on the goat species demonstrating the presence of this characteristic as an influence on reproductive parameters, the prevalence of scrotum bipartition was estimated in the sheep herds reared in the municipality of Patos, Paraiba backwoods, Brazil. A total of 331 rams were examined from farms in four municipalities in the micro-region of Patos, Paraiba, Brazil, and the same study was also carried out at the municipal slaughterhouse in this city, where 456 animals were examined. According to the analysis, 66.67% of the farms visited presented one or more sheep with scrotum bipartition, with a prevalence of 11.48% on the farms and 14.47% at the slaughterhouse. The degree of bipartition was 9.59 ± 1.035% of the total scrotum length for the animals in the field and 12.89 ± 0.749% for those from the slaughterhouse, characterizing bipartition of less than 50% of the scrotum length. The variables intensive rearing (OR = 16.6) and the Dorper breed (OR = 6.91) were identified as factors associated to the presence of scrotum bipartition. It was concluded that scrotum bipartition is a characteristic present in sheep reared in the municipality of Patos in the semiarid region of Paraiba state, northeastern Brazil, and high prevalence was observed of farms with bipartition sheep, but a low number of animals with scrotum bipartition was identified.

## Introduction

The small ruminants, goats and sheep, have great potential to adapt to desert and semiarid regions. Certain morphological characteristics present in these species are signs of adaptation to these environments, related to the process of natural selection, to ensure survival of those animals best adapted to the adverse environmental conditions.

In tropical climate regions, some manifestations have been reported of morphological alterations in the reproductive organs in goats, such as the appearance of division in the scrotum, reported first by Robertshaw (1982) in arid and semiarid regions of East Africa. This characteristic has been very frequently observed in goats reared in northeastern Brazil, and was called by Nunes *et al*. (1983) “bipartitioned scrotal sack”. This anatomy increases considerably the surface of each testicle exposed to ambient temperature, resulting in better heat dissipation, with consequent increase in the testicle biometric parameters, spermatic quality and reproductive efficiency of these goats compared to those without ( Almeida *et al*., 2008 ; 2010 ; Feliciano-Silva *et al*., 1986 ; Nunes *et al*., 1983 ). Other studies have confirmed the efficiency of the bipartition in goat testicular heat regulation ( Lima Júnior and Vianni, 1995 ; Machado Júnior *et al*., 2009 ).

The characteristic of scrotum bipartition has also been observed in sheep in the Morada Nova breed ( Melo *et al*., 2013 ) and in crossbred animals ( Tolentino *et al*., 2014 ). Rodrigues *et al*. (2016) observed difference in the spermatogenic parameters of crossbred sheep with bipartition compared to non-bipartitioned sheep, with higher efficiency in spermatogenesis and Sertoli cell yield in the sheep with scrotum bipartition.

In the Paraiba backwoods (semiarid region of Paraiba state, northeastern Brazil), sheep farmers have also reported the appearance of animals with these characteristics in their herds. Thus, the objective of the present study was to estimate the prevalence of scrotum bipartition in the sheep herds reared in the Paraiba backwoods, Northeastern Brazil.

## Material and Methods

Sheep farms were visited in four municipalities the micro-region of Patos, Paraiba, Brazil (Patos, Santa Terezinha, São José do Bonfim and Quixaba). The same study was also carried out in the Patos municipal slaughterhouse, that receives animals from producers in the town of Patos and nearby towns.

The formula for simple random samples ( Thrusfield, 1995 ) was used to determine the number of sheep farms to be sampled in each municipality, with later correction for finite populations: n = (z^2^.P_esp_. (1-P_esp_ ))/d^2^. Where n = number of farms to be sampled; Z = value of the normal distribution for the 95% level of confidence P = 50% expected scrotum bipartition prevalence (value adopted to maximize the sample); d = sample error 20%.

Based on the farm health records in the archives of the Agricultural Integration System (Sistema de Integração Agropecuária-SIAPEC-PB) of the Secretary of State for Agricultural and Fishery Development (Secretaria de Estado do Desenvolvimento da Agropecuária e Pesca - SEDAP-PB), of the 382 sheep farmers of the four municipalities to be visited, farms were considered for sample calculation that had at least 10 rams, totaling 70 producers. Thus, based on the sample parameters, 13 farms were randomly selected by raffle in the municipality of Patos, eight in Santa Terezinha, three in São José do Bonfim and three in Quixaba, totaling 27 farms.

The sampling methodology described above was followed to determine the number of sheep to be sampled in the slaughterhouse, using 5% sample error, and 456 rams were examined.

On each farm visited, from August 2015 to July 2016, the sheep scrotum were examined to identify the animals that presented scrotum bipartition and determine its degree, that was the ratio between the portion of the scrotum that presented division and the total length. For this, the scrotal length (COE) and the scrotum bipartition size (TB) were measured using a tape measure and a pachymeter, respectively, adapting the proposal by Lima Júnior and Vianni (1995) .

The profile of the population of sheep with bipartition was obtained by applying a questionnaire to obtain information on the rearing system, predominant breeds, animal acquisition and farmers´ knowledge about scrotum bipartition.

The factors associated with the presence of scrotum bipartition were analyzed with the data obtained from the epidemiological questionnaires in two steps, univariate and multivariate analysis. In the univariate analysis the independent variables were submitted to analysis of association with a dependent variable (presence or absence of scrotum bipartition), and those that presented a value P ≤ 0.2 by the chi squared test ( Zar, 1999 ) were selected for multivariate analysis by multiple logistic regression ( Hosmer and Lemeshow, 2000 ). A 5% level of significance was adopted in the multiple analysis and all the analyses were carried out by the SPSS 20.0 program *for Windows*.

The G test was used to compare the bipartition frequencies on farms and in animals among the municipalities and the T student test was used to compare the means of scrotal length, bipartition size and degree of scrotal division between the field and slaughterhouse surveys. A 5% level of significance was adopted and the analyses were made using the BioEstat 5.03 program ( Ayres *et al*., 2007 ).

This study followed the ethical standards for animal experimentation (CEUA/CEP-UFCG Nº 277-2015).

## Results

**Figure 1:**
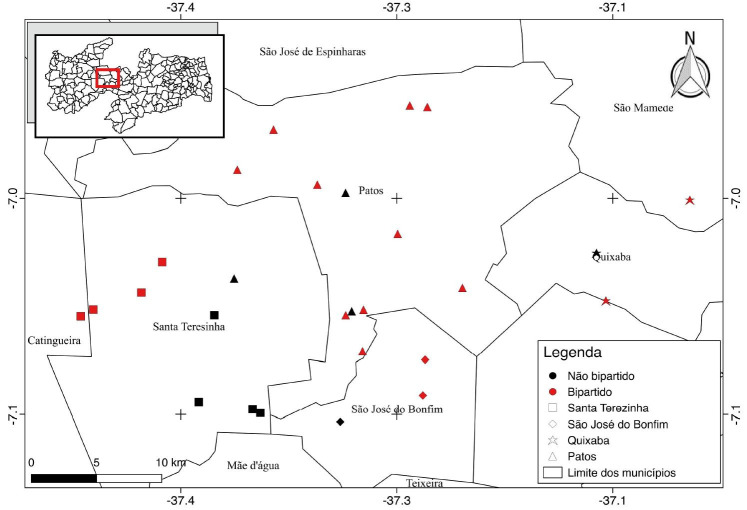
Geographic location of the farms visited in the microregion of Patos, Paraíba backwoods, north-eastern Brazil, according to the presence of animals with scrotum bipartition and without scrotum bipartition. Patos-PB, Brazil, 2017. (Projection: Coordenadas Geográficas/ Datum: SIRGAS2000/ SIG: QGIS 2.14.0-Essen).

According to the data obtained in the present study, 66.67% of the farms visited ( Figure 1 ) presented one or more rams with scrotum bipartition.

Table 1 shows that in the municipalities of Patos, Santa Terezinha, São José do Bonfim and Quixaba, 76.92%, 50%, 66.67% and 66.67% of the farms had bipartitioned rams, respectively, and there was no significant statistical difference among the municipalities (P > 0.05). The prevalence of rams with scrotum bipartition was 11.48% and statistical difference (P > 0.05) was not observed between the municipalities assessed ( Table 1 ). The prevalence of bipartition rams was 14.47% in the animals from the slaughterhouse and there was no statistical difference between the data obtained in the municipalities compared to those of the slaughterhouse (P > 0.05).

**Table 1:** Percentage of prevalence of property with ram with scrotum bipartition and animals with the characteristic in the municipalities of Patos, Santa Terezinha, São José do Bonfim e Quixaba, belonging to the micro region of Patos, Paraíba backwoods, Patos-PB, Brazil, 2017.

Municipalities	Farms		Animals	
	Bipartite	Non bipartite	Bipartite	Non bipartite
Patos	76.92^a^	23.08	11.27^a^	88.73
Santa Terezinha	50^a^	50	9.88^a^	90.12
São José do Bonfim	66.67^a^	33.33	14.81^a^	85.19
Quixaba	66.67^a^	33.33	10.53^a^	89.47

Frequencies followed by the same letter in the same column do not differ statistically by the G test (P > 0.05).

**Figure 2:**
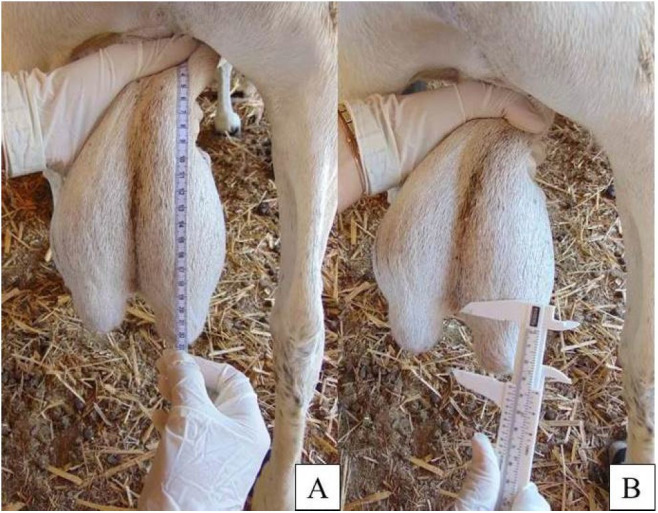
Scrotum bipartition in sheep less than 50% of the scrotum length. (A) Scrotal length and (B) scrotum bipartition length.

Table 2 shows the mean scrotum length, mean bipartition size and scrotum bipartition degree identified in the sheep in the field survey and in the sheep examined in the slaughterhouse. The degree of bipartition was 9.59±1.035% for the animals in the field, that was less than those from the slaughterhouse, that was 12.89 ± 0.749% (P < 0.05), that characterizes bipartition of less than 50% of the scrotal length ( Figure 2 ).

**Table 2:** Degree of bipartition (mean + standard error) in ram. Patos-PB, Brazil, 2017.

	Scrotal length (cm)	Bipartition Size (cm)	Degree of scrotal division (%)
Field surveys	16.67 ± 0.777^a^	1.19 ± 0.177^a^	9.59 ± 1.035^a^
Slaughterhouse	14.70 ± 0.403^a^	1.53 ± 0.125^a^	12.89 ± 0.74^b^

Means followed by the same letter in the same column do not differ statistically by the student t test (P > 0.05).

The following variables were selected in the univariate analysis of factors associated to the presence of scrotum bipartition ( Table 3 ): rearing system, predominant breeds, animal acquisition and farmers´ knowledge on scrotum bipartition. After multivariate analysis by multiple logistic regression, the risk factors identified were intensive type rearing (OR= 16.6) and the Dorper breed (OR= 6.9; Table 4 ).

**Table 3:** Univariate analysis for risk factors associated to scrotum bipartition prevalence in sheep in the municipality of Patos, state of Paraíba, Brazil.

Variables	Category	Total animals	Nº animals bipartites (%)	P
Type of creation	Intensive	8	3 (37.5)	
	Semi-intensive	298	33 (11.0)	
	Extensive	25	1 (4.0)	0.032*
Predominant breeds	Santa Inês	160	15 (9.4)	
	Dorper	12	5 (41.7)	
	Sem Raça Definida (SRD)	149	16 (10.7)	
	Others	10	1 (10.0)	0.008*
Buy animals	Yes	224	29 (12.9)	
	No	107	8 (7.5)	0.197*
Where / from whom	Keeps the flock	107	8 (7.5)	
	Exhibition	18	6 (33.3)	
	Auction / fair	15	2 (13.3)	
	Traders	112	12 (10.7)	
	Others farms	79	9 (11.4)	0.033*
I had already observed the scrotal bipartition	Yes	106	10 (9.4)	
	No	225	27 (12.0)	0.614*
Already had in the breeding herd with bipartition	Yes	6	0 (0.0)	
	No	138	15 (10.9)	
	Did not know how to report	187	22 (11.8)	0.659*
*(P ≤ 0.2)				

**Table 4:** Risk factors associated to scrotum bipartition prevalence in sheep herds in the municipality of Patos, state of Paraíba, Brazil.

Risk factors	Regression coefficient	Default error	Wald	Degrees of freedom	Odds ratio	IC 95 %	P
Intensive breeding	2.810	1.320	4.532	1	16.6	1.3 – 220.9	0.033
Breed Dorper	1.930	0.655	8.675	1	6.9	1.9 – 24,9	0.003

The answers to the questionnaire regarding rearing showed correlation between scrotum bipartition and farms with intensive management. However, 70.38% of the farmers did not know about this characteristic or how to tell whether there was a bipartitioned reproducer in their herds. Bipartition was more prevalent (33.3%) among most farmers who introduced animals in their herds (70.38%), and especially amongst those who acquired animals at livestock shows (10.53%) compared to the farms that did not acquire animals from outside sources where only 7.5% of the total of animals had scrotum bipartition ( Table 3 ). Bipartition was also the observed in Santa Inês sheep (9.4%) and in crossbred sheep (10.7%; Table 3 ).

## Discussion

Studies on goats show that the scrotum of this species can present different degrees of bipartition; in addition to animals without bipartition, two degrees of bipartition were established, grouping animals with scrotum division shorter than 50% of the total scrotum length and those with division longer than 50% ( Almeida *et al*., 2008 ; 2010 ; Feliciano-Silva *et al*., 1986 ; Machado Júnior, 2009; Nunes *et al*., 1983 ; Nunes *et al*., 2010 ; 2013 ), unlike observations in sheep, where only animals with bipartition less than 50% of the total scrotum length were identified.

Previously, due to lack of knowledge, the fact that an animal presented a division in the scrotum was considered a defect by most farmers ( Smith and Sherman, 2009 ), but, due to the importance of this characteristic for the animals, a finding in several studies, both in goats ( Almeida *et al*., 2008 ; 2010 ; Feliciano-Silva *et al*., 1986 ; Machado Júnior *et al*., 2009 ; Nunes *et al*., 1983 ) and sheep ( Rodrigues *et al*., 2016 ), goat and sheep rearing associations began to accept bipartition in reproducer selection. An acceptable degree of bipartition for each breed standard is determined and it is understood that those who do not mention this characteristic, do not prohibit it (Associação Brasileira de Criadores de Ovinos, Portal do Boer, 2018; Associação Brasileira de Criadores de Caprinos, 2018).

More sheep with scrotum bipartition were observed on farms with intensive management, that may be related, according to Gouveia *et al*. (2007) , to the fact that farms with intensive management use more technology and make better animal selection, where the farmers invest in in acquiring animals to improve the herd, introducing bigger genetic variability in the production, although this selection is not being made in function of the characteristic studied, since most farmers do not know about scrotum bipartition.

However, it can also be considered that, although most sheep farmers did not know about this characteristic, the fact that it is a morphological adaptation to high temperatures ( Nunes *et al*., 1983 ), animals would be increasing bipartition development and passing it to their progeny. This suggests that this morphological adaptation might be linked to selection of improved animals and reinforces the theory of a rearing system with selected animals.

Other data that may reinforce this theory is the Dorper breed as a risk factor for the appearance of animals with bipartition because they are animals better adapted to high temperatures, presenting high rusticity and adaptability ( Milne, 2000 ). According to Mendes (2014) , Dorper sheep can maintain the body temperature within the normal physiological variation, avoiding hyperthermia in high-temperature situations.

Besides the Dorper breed, crossbred sheep presented scrotum bipartition, that was also observed by Tolentino *et al*. (2014) and Rodrigues *et al*. (2016) , and in goats, where crossbreds predominated in the studies on bipartition ( Almeida *et al*., 2008 ; 2010 ; Feliciano-Silva *et al*., 1986 ; Machado Júnior *et al*., 2009 ; Nunes *et al*., 1983 ). This fact may be associated to the predominance of animals raised by breed crossings with native species to increase rusticity in regions with arid or semiarid climates.

It was concluded that scrotum bipartition is a characteristic present in the sheep reared in the municipality of Patos in the Paraíba backwoods, semiarid region. A high prevalence of farms was observed with rams with bipartition, but few animals with bipartition were identified per farm. The degree of the scrotum division did not reach 50% of the total length, and was less pronounced than that observed in goats. The variables associated with the presence of scrotum bipartition in the herd were intense rearing type, with bigger presence of animals with bipartition and the breed, and the Dorper breed presented the highest percentage. It can be suggested that scrotum bipartition in sheep seems to be a characteristic that is still developing in this species.
